# A User-Centered Approach to Achieving High Degree of Digital Health Technology Utilization for Community HIV Case Management and Data Collection in Ethiopia

**DOI:** 10.9745/GHSP-D-24-00353

**Published:** 2026-08-10

**Authors:** Legese A. Mekuria, Getaneh Likasa, Temesgan Sintayehu, Leul Mekonen, Asayehegn Tekeste, Endris Seid, Wondwossen Asefa, Afework Negash, Abiy Shewarega, Mesfin Tilaye, Gizachew Eyassu, Kidist Belete, Mohamed Nur, Steven Neri, Adrienne Hayes, Emily Liddell, Sangeeta Mookherji, Dawit A. Tsegaye

**Affiliations:** a Project HOPE, Addis Ababa, Ethiopia.; b Project HOPE, Washington, DC, USA.; c Previously with USAID, Addis Ababa, Ethiopia.; d U.S. Embassy, Foreign Assistance Section, Addis Ababa, Ethiopia.; e Project HOPE, Windhoek, Namibia.

## Abstract

Digital health technologies have untapped potential to transform community health systems, and they are underutilized for chronic HIV care and electronic data management in low- and middle-income countries. We describe the design, development, and use of a mobile health application in community-based HIV prevention, care, and treatment programs implemented in Ethiopia since 2017. Initially, these programs utilized aggregate data collection methods, which were fragmented, non-uniform, and largely paper-based, posing challenges to data quality and data use. Local software developers applied user-centered methods to build a Unified Data System (UDS), an innovative digital health solution aimed at standardizing case management and routine data collection procedures. Developers involved end-users, including frontline community health workers (CHWs) and program managers, in the design and development process. They also conducted field visits to understand and assess user activities and needs. Developers and program staff conducted field testing, and they made design changes iteratively to incorporate user feedback. Local implementing partners deployed 950 CHWs to use the UDS for HIV service delivery and client-level data collection offline. Data were stored centrally in the CommCare HQ web service backed by a local analytics server. The UDS was linked to Power BI for advanced data analytics and visualization. Data validation tests were performed prior to indicator computation and reporting to the next level. 1,766 mobile accounts were opened for CHWs, of which 1,628 (92.2%; 95% confidence interval [CI]=90.8%, 93.4%) have submitted data. An additional 368 of 392 web accounts (93.9%; 95% CI=91.0%, 96.0%) have been used by program managers and monitoring and evaluation experts to access the dashboard. The UDS has eliminated the tedious, manual, paper-based work associated with data (dis)aggregation and reporting by frontline CHWs and local implementing partners. Hence, the number of days to access client-level data by the local partners was reduced from 1 month to just 1 day, and the time to report on activity performance was shortened from 5 days to a half-day. Data completeness was nearly 100% and data validation tests demonstrated no invalid data elements or errors. Generally, there was a high degree of digital health technology utilization by frontline CHWs and local implementing partners for standardized HIV care, high-quality data collection, and making data-informed decisions. Lessons learned from the UDS implementation could be adapted not only to support community HIV programming but also to strengthen national electronic community health information systems.

## Abstract (precis)

A mobile health application, co-designed with frontline CHWs and local partners, enabled standardized HIV case management and high-quality, real-time community data collection, analysis, and use that informed program decision-making.

## Teaser

**Key Findings:**

- A user-centered mobile health application, co-designed with frontline community health workers and local partners, was successfully deployed across community HIV programs in Ethiopia, supporting standardized case management and client-level data collection.
- High degrees of utilization and data quality were achieved: over 92% of the mobile accounts were used by frontline workers and 94% of the web accounts by implementation managers, with close to 100% data completeness scores for key indicators and no invalid data elements detected.
- The number of days to access client-level data by local partners was reduced from 1 month to 1 day, and the time to report on activity performance from 5 days to half a day.
- Iterative field testing, training, and routine technical assistance facilitated acceptability and sustained use despite a longer-than-expected implementation timeline.

**Key Implications:**

- The digital health initiative showed the feasibility of designing, developing, and using digital tools for electronic case management and high-quality data collection in community settings of a low-income country in Africa.
- Developers should involve end-users in the design and development process, incorporate user feedback, and provide training and routine technical assistance to achieve high levels of usage.
- Continued investment in training, technical support, local capacity development, and iterative updates is needed to sustain digital health technology utilization and to scale similar solutions in comparable low- and middle-income country contexts.

## INTRODUCTION

The World Health Organization (WHO) has defined 6 essential components of a strong health system comprising health workforce, health service delivery, health information system, access to essential medicines/vaccines/technologies, health financing, and leadership and governance.^1^ These components are crucial for expanding access to health care and for supporting public health policy and practice for improved health outcomes. Digital health comes of age to strengthen the health system of low- and middle-income countries in its broader scope.^2^^–^^5^

Current literature defines digital health as the knowledge and activities combining health care and information and communication technology.^6^^,^^7^ According to the U.S. Food and Drug Administration (FDA), “digital health includes mobile health (mHealth), health information technology, wearable devices, telehealth and telemedicine, and personalized medicine.”^6^ Digital health serves as an electronic platform from which essential health services are delivered in an integrated approach and client-level data are collected, managed, or processed in real-time.^2^^,^^3^

In low-income countries, such as in Ethiopia, digital health has the potential to empower community health workers (CHWs) for coordinated and efficient health service delivery. It has a role in addressing major challenges in the national health management information system (HMIS), including poor data quality and lack of data-driven action at all levels.^5^^,^^8^ With the increasing number of mobile phone subscribers and the rapid expansion of high-speed internet technology,^9^^,^^10^ several new initiatives on digital health applications and tool development are underway.^11^^,^^12^ This has created enormous opportunities for digital health technologies to empower health workers, support case management, improve service quality, and strengthen the community health information system at large.^11^^,^^12^

Previous studies showed that digital health interventions have helped people self-manage chronic conditions, such as diabetes,^13^^,^^14^ cardiovascular diseases,^15^^,^^16^ and HIV/AIDS.^17^^,^^18^ Studies conducted in Kenya,^19^ Uganda,^20^ and Nigeria^21^ showed positive outcomes of short message service (SMS) delivery for treatment reminders to improve adherence. In addition, digital tools facilitated the health insurance claims management system,^22^^,^^23^ strengthened stock management,^24^ improved essential health services coverage,^25^^,^^26^ and supported the rapid response to pandemics in Africa.^23^

Digitally enabled health information systems have been used to collect high-quality data,^27^ especially in community settings where there is no or limited internet access.^28^ The data gathered through digital tools can be analyzed to gain further insight into performance gaps or unmet needs, address quality issues, and conduct data-driven decision making.^24^^,^^26^^–^^28^ Digital health technologies also have been used to strengthen national community health information systems, such as the national electronic community health information system (e-CHIS) of Ethiopia.^11^^,^^24^ In the health facilities of Ethiopia, electronic medical record systems, such as SmartCare, are designed to facilitate HIV service delivery and patient data management. Data are extracted from SmartCare and manually entered to DHIS2 for aggregate reporting. However, there is lack of interoperability between e-CHIS (community-level data), SmartCare (facility-level data), and DHIS2 (national aggregate data reporting system). Digital health could play a crucial role in linking community- and facility-based health services and standardizing health care delivery and data management procedures.^26^

In Ethiopia, the U.S. President’s Emergency Plan for AIDS Relief (PEPFAR) supports a community HIV care and treatment (CHCT) activity; comprehensive HIV prevention, care, and treatment for key and priority populations (KPPs); and programs on caring for orphans and vulnerable children (CVC). Initially, individual client-level services were not tracked over time nor were they integrated. Data management procedures were not streamlined and had used non-uniform, largely paper-based, and aggregate data-gathering tools posing challenges to data quality and data use. Software developers at Project HOPE applied user-center design principles^29^^,^^30^ to build the unified data system (UDS), which is an innovative digital health solution aimed at standardizing the workflow and unifying the data collection, analysis, and reporting procedures of CHCT, KPPs, and CVC.

The use of digital health technologies to support large-scale community-based HIV programs is a relatively recent development that has not been assessed in Ethiopia. In this implementation research, we describe the design, development, and deployment process of the UDS to support innovative approaches of case management in the community-based HIV prevention, care, and treatment activities. We also describe potential reasons for the high degree of digital health technology utilization by frontline CHWs and local implementing partners to collect client-level health information and conduct data analysis, interpretation, and use to inform community HIV programming.

## METHODS

### Project Description and Setting

In Ethiopia, PEPFAR-supported community HIV programs have been implemented since 2017 in high HIV prevalence areas of Addis Ababa, Amhara, Oromia, Southern Nations, Nationalities, and Peoples (SNNP) (which includes newly formed regions such as Central Ethiopia and South Ethiopia), Sidama, South-West Ethiopia People (SWEP), Tigray, and Gambella regions. Six prime local implementing partners that are legally registered in Ethiopia—Mekdim Ethiopia National Association (MENA), Amhara Development Association (ADA), Integrated Service on Health and Development Organization (ISHDO), Mary Joy Ethiopia (MJE), Love In Action Ethiopia (LIAE), and Beza Posterity Development Organization (BPDO)—signed a cooperative agreement with the U.S. Agency for International Development (USAID), and 25 local sub-grantees signed service-level agreements with prime implementing partners to deliver community-based HIV services. Project HOPE, Population Services International (PSI), and FHI 360 provided technical assistance to CHCT, KPP, and CVC projects, respectively.

Project HOPE partnered with 16 local implementing partners to provide index case contact testing, HIV self-testing, community-enhanced adherence counseling, differentiated service delivery, care and treatment, and the tracing of interruption in treatment cases in high HIV prevalence areas. Similarly, PSI and 5 other local implementing partners implemented the KPP project, which targeted vulnerable populations, such as female sex workers (FSWs) and their sexual partners. The KPP project delivered HIV testing, pre-exposure prophylaxis (PrEP), and antiretroviral therapy (ART) services in drop-in centers and/or in community service delivery points (SDPs) as outreach. Furthermore, FHI 360 implemented the CVC project. It supported the government of Ethiopia and local partners to deliver services related to health, education, nutrition, economic security, protection, and psychosocial well-being of orphans, caregivers, and families who are infected with or affected by HIV.

These programs deployed frontline CHWs such as community engagement facilitators (CEFs), social service workers, nurses, and data clerks who had received 2 to 4 years of professional training. They were members of the local community who were chosen by the community (or local implementing partner) to provide quality HIV services.^31^ Volunteers, including community response persons and case workers with primary-level education, supported CEFs and social service workers, respectively. Case managers who work in nearby health facilities were involved in line-list sharing and patient referral with CHWs. CHCT, KPP, and CVC projects targeted vulnerable and high-risk populations to achieve the national 95-95-95 HIV treatment targets.^32^ These projects were using fragmented, largely paper-based, and aggregate data collection and reporting systems, posing challenges to data quality and data use. Therefore, developers at Project HOPE built a UDS to overcome these problems.

### Unified Data System

UDS is an innovative digital health solution designed to streamline individual case management processes, empower health workers and stakeholders with data gathering, analysis, reporting, and insightful visualization tools, and help them make data-driven decisions. UDS was built on the CommCare digital platform,^33^ chosen for the following reasons:

- Open source, thus easily customized to support local language and calendar
- Has robust offline case management and data gathering functionality
- Interoperable with the national e-CHIS, which is also built on the same type of platform
- Suitable in resource-limited settings with low internet bandwidth

CommCare is an Android application framework designed for Java or Android mobile devices (mainly smartphones and tablets) with low storage capacity.^33^ It was used to digitize software modules tailored to standardize case management across distinct programs, including CHCT, KPP, and CVC.

### UDS Workflow Design and Development

UDS developers applied user-centered design principles including user focus (and their needs), user involvement, user experience or feedback, and iterative design throughout the development process.^29^^,^^30^ User-centered design was chosen because previous studies suggested that it could potentially lead to a high degree of acceptability and utilization by target users.^34^^–^^36^

Therefore, developers conducted desk review of documents and forms to understand core activities by frontline CHWs and to assess the key roles and responsibilities of stakeholders. They also conducted a scoping field visit to observe client journeys, assess users’ needs, and gather the requirements for workflow design. In addition, developers interviewed clients, officials at the Ministry of Health, and end-users (i.e., frontline CHWs and implementation managers of local implementing partners) to define the full scope and desired functionality within the app. Furthermore, developers assessed client health information flow between health facilities and communities and from SDPs to the CommCare data repository. User feedback was collected onsite during field visits and review meetings, as well as remotely via telephone, e-mail, virtual sessions, rapid assessments, and UDS partners’ fora. Developers and program staff also conducted user acceptance testing among CEFs, case managers, and program managers in selected community SDPs, health facilities, and local implementing partner offices, respectively.

### UDS Architectural Design

UDS architecture constitutes various independent software components for streamlined client-level data collection all the way to reporting and visualization. The architecture leverages industry standard API (Application Programming Interface) technologies to enable loosely coupled system integration, effectively enhancing the maintenance and scalability of the overall system.

**FIGURE 1:**
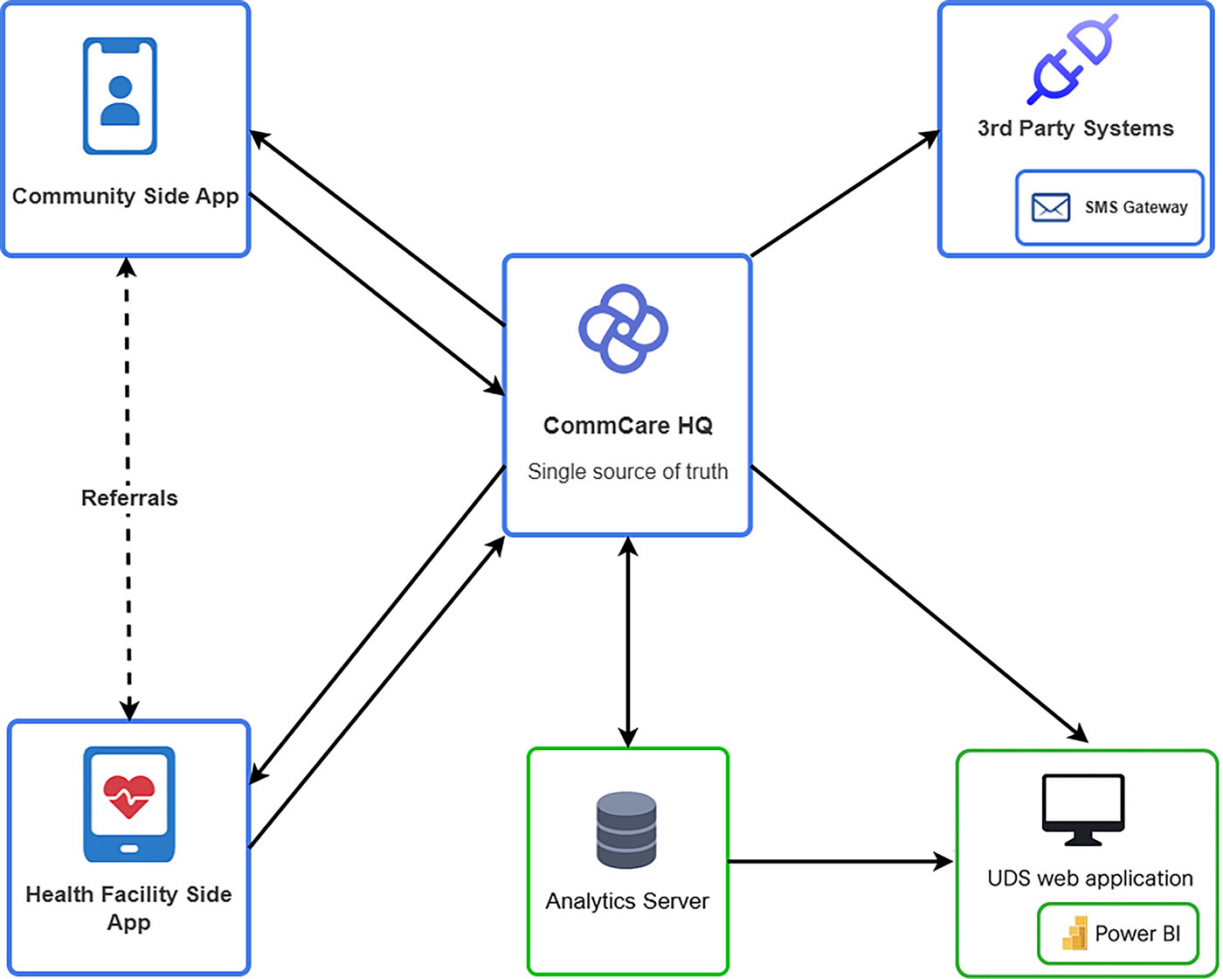
High-Level Architectural Design of the Unified Data System Software Components, 2024

UDS has 4 major software components (Figure 1):

1. The CommCare mobile app, which was installed on smartphones or tablets enabling frontline CHWs to collect client-level data in real time.
2. The CommCare HQ web service, which serves as the central data repository that collects and stores the raw client-level data transmitted from the mobile application. The CommCare HQ web service also has capabilities to interface with other external software systems, such as the SMS gateway of Twilio^37^ for text messaging.
3. The local analytics server, which sits between the CommCare web service and Power BI, orchestrates as a data pipeline and uses advanced algorithms to run preliminary data analysis and data cleaning routines before passing the cleaned data to the next stage of data visualization. Moreover, this layer exposes high performance API end points to other third-party systems such as Power BI and DHIS2, eliminating technical data bandwidth limitations observed in CommCare repository endpoints.
4. The processed and refined data from the analytics server is fed into Power BI, which is the visualization powerhouse of the UDS. The UDS Power BI transforms raw datasets into interactive dashboards generating visuals of key performance indicators (KPIs) to enhance data use for planning, monitoring, and making decisions across all programs.

### Digital Case Management Framework

The UDS digital case management framework was adapted from prior research and PEPFAR-supported programs^38^^,^^39^ and modified to suit the local context. We defined case management broadly as the process of client identification, need assessment, planning, person-centered HIV care, and follow up over time and across places to achieve desired health needs or outcomes.^38^^,^^39^ UDS implemented a user-friendly mobile application containing digital case management modules for the PEPFAR community HIV prevention, care, and treatment activities.

**FIGURE 2:**
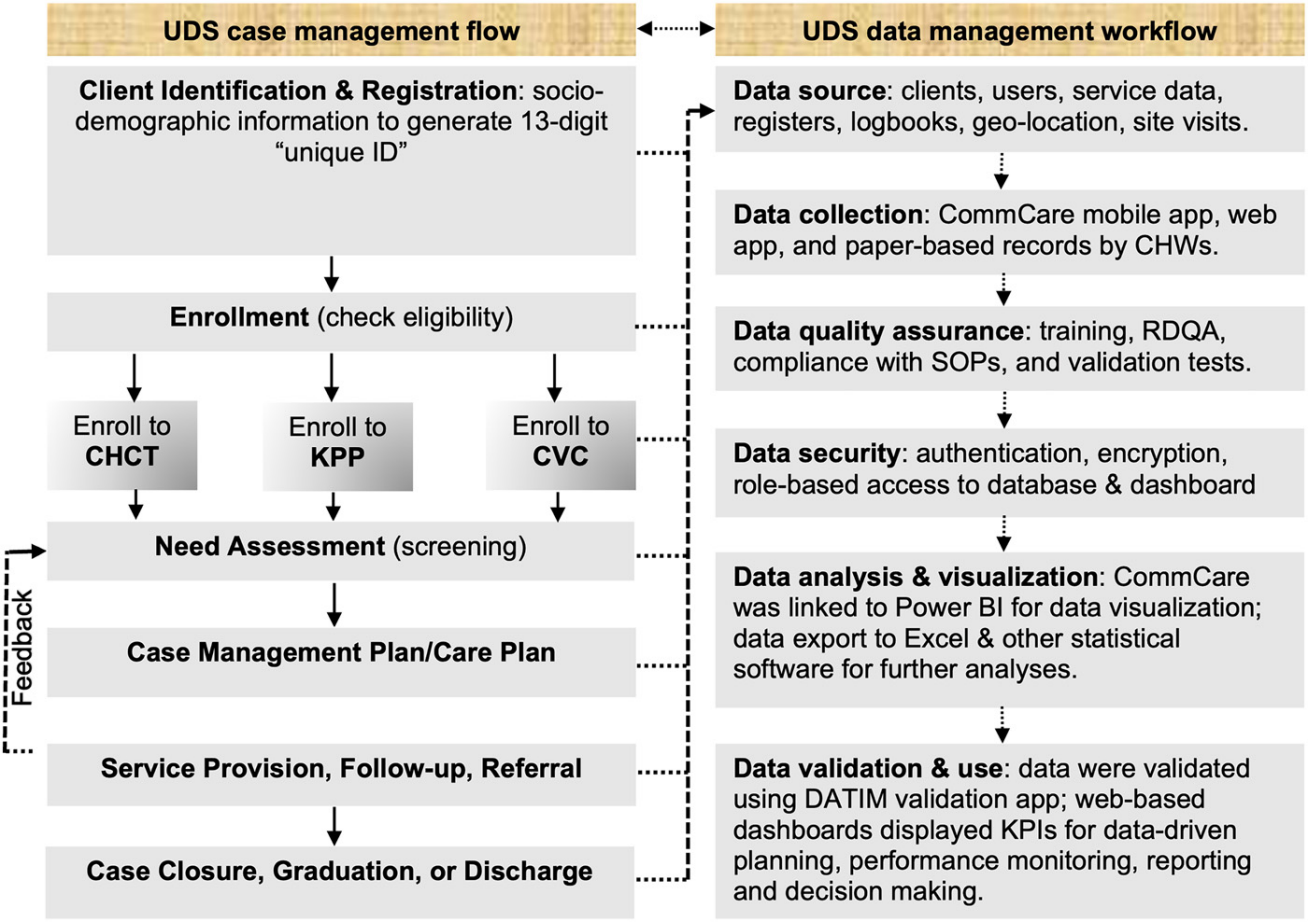
Use of the UDS as a Digital Case Management Platform and Data Management Tool in the PEPFAR-Funded Community HIV Activities in Ethiopia, 2024 Abbreviations: CHCT, community HIV care and treatment activity; CHW, community health worker; CVC, caring for orphans and vulnerable children; DATIM, data for accountability, transparency, and impact; KPI, key performance indicators; KPP, comprehensive HIV prevention, care, and treatment for key and priority populations; RDQA, routine data quality assessment; SOP, standard operating procedure; UDS, Unified Data System.

Digital case management navigated the stages of client identification and registration, need assessment, automatic care plan generation, service delivery and follow up, referral, graduation assessment, and case closure ensuring comprehensive health care delivery (Figure 2). A registration form was used to register clients and enroll them in one of the specified programs. This module was the entry layer, capturing essential client-level demographic information to generate a 13-digit “unique ID” and initiate the case management process. The unique ID helped frontline CHWs to manage client encounters at the SDP controlling for duplicates. Another 36-digit unique ID was generated by the CommCare web system, which we used mainly for client tracking, data merging, and analysis.

Following successful enrollment, the next step was screening or need assessment. Then, UDS software generated an automatic care plan based on the information entered in the need assessment form. The automatic care plan generation was a crucial step to ensure that each client received personalized care. This module encoded service delivery data elements. Whether it was medical services, referral, counseling, or educational sessions, the software ensured that the service delivery was precisely aligned with the generated care plan. User interactions, delivery of services, and client responses were systematically fed into the application, enabling the software to adapt and refine care plans to the ever-evolving client needs. As clients progressed through the iterative care plan and service delivery process, the application assessed their eligibility for graduation. Upon achieving the predefined milestones and meeting the graduation criteria, the system marked the case for closure or discharge, signifying the successful completion of the case management journey. UDS case management flow also incorporated a referral module, which facilitated digital line-list sharing and bidirectional referral between community and health facility settings.

### UDS Data Management Procedures

#### Data Collection and Storage

**FIGURE 3:**
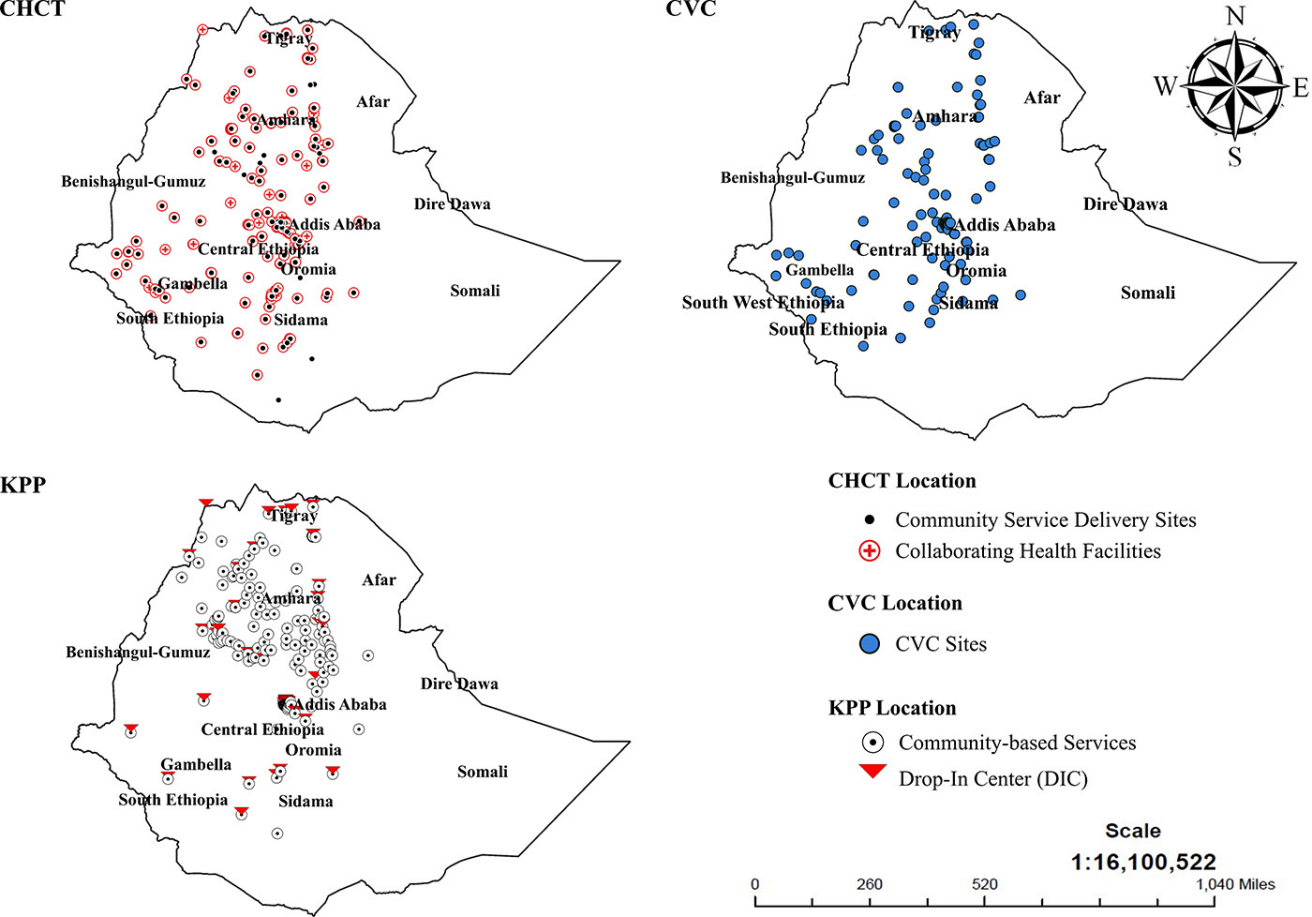
Illustrative Geo-Referenced Map Depicting Service Coverage and Utilization Based on UDS Mobile App Data in the PEPFAR-Funded Community HIV Activities in Ethiopia, 2024 Abbreviations: CHCT, community HIV care and treatment activity; CVC, caring for orphans and vulnerable children; KPP, comprehensive HIV prevention, care, and treatment for key and priority populations; UDS, Unified Data System; DIC, Drop-in center.

Frontline CHWs used a mobile application to collect client-level data at the SDP level. Routine data collected at this level served as the building block of UDS data management, establishing the foundation for subsequent data processing. Client-level data were recorded on paper-based registers and entered to the UDS system offline. Submitted forms were synchronized to the server when internet connectivity was available. CommCare HQ web service^33^ was the central data repository, storing the raw client-level data transmitted from the mobile application, and it was backed by a local server. It was within CommCare HQ that raw-level data reports were generated for various thematic programs. UDS also supported the collection and storage of geo-locations such as latitude and longitude of SDPs. These data were used to develop geo-referenced maps, which depict service coverage and utilization in the PEPFAR-supported areas of Ethiopia (Figure 3).

#### Data Security, Privacy, and Confidentiality

Multiple security layers including basic authentication (username and password) and role-based access to client data were in place. Users were required to change the login information regularly. In addition, client data were entered and stored encrypted-at-rest (symmetric AES256), and data were transferred from mobile devices to the server (and vice versa) through a secured and encrypted HTTPS channel.^33^ Moreover, Dimagi has put in place multiple security layers including at the operational level (physical data centers and servers), mobile application level, user level, and transmission/network level.^33^

#### Data Quality Assurance

A standard operating procedure was in place to ensure compliance with high-quality data collection standards (Supplement 1). Basic and refresher training was provided for frontline CHWs. Validation logic was built to prevent incorrect or incomplete data entry. Routine data quality assessment and site-level data verification visits were done to cross-check data in the UDS with data on the paper-based registers. Real-time data entry tracking dashboards were developed (on Power BI) to monitor timely data entry. In addition, the monitoring and evaluation officers routinely checked the data for timeliness, completeness, and accuracy. Regular data cleaning practices, such as de-duplication of multiple entries, were done monthly. Finally, data validation tests were performed using a data for accountability, transparency, and impact (DATIM) validation tool prior to indicator computation and reporting to the next level.^40^

#### Data Analysis and Visualization

UDS was linked to Power BI software^41^ for advanced data analyses, KPI computation, and visualization on a dashboard. The UDS Power BI transformed raw datasets into interactive dashboards generating visuals of reportable indicators and KPIs according to the PEPFAR monitoring, evaluation, and reporting guide.^42^ Web-based dashboards were used for tracking progress, reporting, and gaining insight.

Most KPIs were expressed in numerical figures, for example, “the number of individuals who were identified/tested using index HIV testing services and received their results.”^42^ Other indicators were computed as percentage, such as “the proportion of adults and pediatric patients on antiretroviral treatment with suppressed viral load results in the past twelve months.”^42^ All the indicators were disaggregated by age, sex, and geographic location, and they were visualized in tables, bar charts, pie charts, or line graphs. Data were exported to Microsoft Excel^43^ and Stata^44^ for descriptive analyses.

### Outcome Measures

The main outcome measures were UDS utilization, data access and availability, data validation checks, and data completeness:

- **UDS utilization**: expressed as the percentage of mobile accounts used by frontline CHWs to submit client-level health service data. A related outcome measure was the proportion of web accounts used by program managers to access the dashboard for reviewing performance. Operationally, we defined an active mobile or web account if the user had logged in and/or submitted data at least once within 6 months prior to December 31, 2024, or at least once within 6 months immediately before the date the account was closed (or had become inactive). Account status was established at the recorded date of login activity, the last known date of login, or the final study date (December 31, 2024).
- **Data access and availability**: defined as the number of days that elapsed between client-level data collection (by CHWs) and data access by local implementing partners. An additional outcome measure was the time interval between data analysis and reporting to the next level by local implementing partners.
- **Data validation checks**: expressed as the percentage of data elements or values that were valid or contained no errors. Data were considered valid with respect to the location where data were collected; alignment with PEPFAR indicator definition, service type, disaggregation, and reporting period; and showed no duplication.^40^
- **Data completeness composite score**: expressed in percentage as the average of all the required data points completed without any missing values. For priority activities, such as HIV testing services, we checked the number of data elements completed with values. Values that were available were scored as 1 and missing values were scored as 0. Then, the average composite score was calculated in percentage as the number of complete data elements divided by the total number of data elements checked, multiplied by 100. The minimum composite score was 0% (0 complete out of 7 checks) and the maximum possible score was 100% (7 complete out of 7 checks), with a higher composite score indicating more complete data.

## RESULTS

### UDS Platform Design and Development

UDS platform establishment began in 2018 when 3 software developers, over 11 HIV care and treatment program managers, health care workers, monitoring and evaluation experts, and 6 local implementing partners participated in the design and development process. Developers applied user-centered design principles including user focus, identifying user needs, incorporate user feedback, and iterative design and development.

#### Understanding User Activities

We conducted 3 scoping visits in 7 urban and semi-urban community-based service delivery sites in Addis Ababa, Bahir Dar, and Bishoftu towns. Developers held a follow-up field visit to 4 service delivery sites in Addis Ababa, Adama, Bishoftu, and Gambella towns, which included on-site observation and interview with 7 CHWs and 3 local implementing partners.

#### Identifying User Needs

During the field visit, frontline CHWs expressed their interest in using a digital system to register and manage clients, finding them easily from a case list, and reporting on the number of clients with disaggregation by age, sex, geography, and service type. Similarly, local implementing partners said they needed daily data access to monitor activities, review performance, and develop reports electronically. Based on our observation, both CHWs and local implementing partners seemed eager to transition fully from a paper-based to digital system.

#### Prototyping Design and Development

Developers sketched out programmatic workflows, content, and technical design of the app during the field visit. They digitized 98 paper-based forms or modules in the CommCare mobile platform, of which 63 (64%) belonged to CHCT, 18 (18%) were KPP, 13 (13%) were CVC, and 4 (4%) were common to the 3 programs. Then, developers built a mobile application that mirrored the key registration and client management processes required by CHCT, KPP, and CVC.

An organizational hierarchy that allowed case sharing and data review across CHWs within an SDP was developed with 7 administrative regions and Addis Ababa city administration at the highest level. A total of 373 sub-national units or districts were formed beneath the top level, and 950 SDP sub-levels were established further down the hierarchy. Moreover, 950 frontline CHWs were assigned to specific SDPs based on their community work locations.

#### Field Testing

Developers conducted user acceptance testing in 7 purposively selected SDPs in Addis Ababa, Adama, and Gambella towns. They sought user feedback from 9 CHWs and program managers at 3 local implementing partners. Information gathered from these end-users helped developers to revise and improve the UDS system’s functionality, acceptance, and ease of use by frontline CHWs (Supplement 2).

The first fully functional version of the mobile application was rolled out in February 2020. Developers added new modules and functionalities, such as SMS reminders, geolocation data capture, and data validation checks thereafter. After the initial rollout, developers received user feedback that prompted changes to the mobile app. To date, 397 updated versions of the mobile app have been released, and all version control is managed centrally by the developers. Reasons for version update included program modification and tool revision, evolving needs from target users, software maintenance or bug fixes, adding new features and modules, user feedback, and making the application more user-friendly.

Each frontline CHW was equipped with a smartphone featuring 2GB to 4GB of RAM, phone charger, and local SIM card from Ethio Telecom. Because smartphones had limited memory space, developers split the organizational units of 10 SDPs with over 6,000 target beneficiaries to better accommodate beneficiary load and to seamlessly transfer data between the central repository and smartphones.

### UDS Deployment and Utilization

UDS was deployed in 950 sites including in 707 (74.4%) community-based SDPs, 201 (21.2%) collaborating health facilities, and 42 (4.4%) drop-in centers. All were found in 373 high prevalence sub-national units or districts of Addis Ababa, Amhara, Oromia, SNNP, Tigray, Sidama, SWEP, and Gambella regions, with one-third (n=317) of the sites in Amhara region followed by Addis Ababa (n=309, 32.5%) and Oromia (n=177, 18.6%) (Table 1).

**TABLE 1.** Characteristics of UDS Community-Based Service Delivery Points, End-Users, and User Accounts as of December 31, 2024, Ethiopia

Characteristic	No. (%)
**Type of SDP**^a^ **(N=950)**	
Community SDPs	707 (74.4)
Collaborating health facilities	201 (21.2)
Drop-in centers	42 (4.4)
**Region of SDP (N=950)**	
Amhara	317 (33.4)
Addis Ababa	309 (32.5)
Oromia	177 (18.6)
Tigray	56 (5.9)
Gambella	31 (3.3)
SNNP	29 (3.1)
Sidama	19 (2.0)
SWEP	12 (1.3)
**Program area of SDP (N=950)**	
CHCT^b^	475 (50.0)
CVC^b^	199 (21.0)
KPP	276 (29.1)
**UDS user accounts ever created (N=2,158)**	
Mobile accounts	1,766 (81.8)
Web accounts	392 (18.2)
**UDS accounts in use as of December 31, 2024 (N=1,407)**	
Mobile accounts	1,066 (75.8)
Web accounts	341 (24.2)
**Type of UDS app end-users as of December 31, 2024 (N=1,291)**	
SDP-level users^a^	950 (73.6)
Above-site users	341 (26.4)
**Sex of UDS app end-users as of December 31, 2024 (N=950)**	
Female	548 (57.7)
Male	402 (42.3)
**Program area of UDS app end-users**^a^ **(N=950)**	
CHCT	579 (60.9)
CVC	264 (27.8)
KPP	107 (11.3)
**Highest educational level of UDS app end-users (N=950)**	
Bachelor’s degree	551 (58)
Diploma	237 (25)
Master’s degree	71 (7.5)
Completed high school	59 (6.2)
Other	32 (3.2)

Abbreviations: CHCT, community HIV care and treatment activity; CVC, caring for orphans and vulnerable children; KPP, comprehensive HIV prevention, care, and treatment for key and priority populations; SDP, service delivery point; SNNP, Southern Nations, Nationalities, and Peoples; SWEP, South-West Ethiopia People; UDS, Unified Data System.

^a^ The relationship between SDPs, community health workers (CHWs), and user accounts is characterized by a many-to-many relational complexity because a single CHW may manage multiple accounts across various SDPs, while a single SDP may be supported by multiple health workers. The number of CHWs and user accounts is also changing due to activation, deactivation, and reactivation of accounts based on workload, attrition, recruitment, or shifting geographic locations.

^b^ Community SDPs could provide both CHCT and CVC services.

As of December 2024, a total of 787 mobile devices equipped with a phone charger and an Ethio Telecom SIM card were in use by frontline CHWs, case managers, nurses, and data clerks. The average top-up credit covering voice calls, internet, and SMS text was Ethiopian Birr 300 for each CHW per month.

Results from two rounds of rapid online assessments by the developers (in June 2022) and by the donor (in July 2024) demonstrated that all local implementing partners fully adopted the UDS system. Program managers also said that UDS satisfied their needs and expectations. Summary results of these assessments can be found in Supplement 3 and Supplement 4.

### User Accounts and End-Users

There were 2,158 user accounts of which 1,766 (81.8%) were mobile and 392 (18.2%) were web accounts (Table 1). At the time of this implementation research, 1,066 (60.4%) mobile accounts and 341 (87%) web accounts were actively in use by frontline CHWs and program implementation managers, respectively.

UDS end-users are broadly categorized into two main groups: users at the SDP level and above-site users. There were 950 users at the SDP level, including 681 (71.7%) frontline CHWs (CEFs, social service workers, nurses, and data clerks), 68 (8%) health care providers in drop-in centers, and 201 (21.2%) case managers or ART focal persons located in collaborating health facilities. Above-site users included 341 program implementation managers or monitoring and evaluation experts working in partner organizations.

Frontline CHWs actively used mobile accounts to register clients, facilitate person-centered HIV care, and collect client-level health service data at the community SDP or drop-in center level. Case managers and ART focal persons used mobile accounts for sharing line-lists and referring clients between health facilities and communities electronically. Program managers used web accounts to monitor service delivery, review performance, generate reports, and access the dashboard for actionable insights.

### Digital Case Management

As of December 31, 2024, CHWs registered 2,858,427 people, including people living with HIV, sexual partners, children, guardians, and other KPPs, in UDS as beneficiaries. More than half (59.2%) (n=1,691,292) were females (Table 2).

**TABLE 2.** Characteristics of People Registered in UDS as Beneficiaries as of December 31, 2024, Ethiopia (N=2,858,427)^a^

Characteristics	No. (%)
**Sex**	
Female	1,691,292 (59.2)
Male	1,167,135 (40.8)
**Program area**	
CVC	1,284,220 (44.9)
KPP	977,066 (34.2)
CHCT	597,141 (20.9)
**Region of residence**	
Amhara	1,123,700 (39.3)
Addis Ababa	790,556 (27.7)
Oromia	558,888 (9.6)
SNNP	145,024 (5.1)
Sidama	80,110 (2.8)
Tigray	79,341 (2.8)
Gambella	54,045 (1.9)
SWEP	26,763 (0.9)

Abbreviations: CHCT, community HIV care and treatment activity; CVC, caring for orphans and vulnerable children; KPP, comprehensive HIV prevention, care, and treatment for key and priority populations; SNNP, Southern Nations, Nationalities, and Peoples; SWEP, South-West Ethiopia People; UDS, Unified Data System.

^a^ Total number of forms submitted=20,336,335.

Digital case management also encompassed line-list sharing and client referral between health facilities and communities electronically. Between FY2020 and FY2024, case managers registered a line-list of 46,195 clients with missed appointments at the health facility. They assigned CEFs to start tracing and follow-up in the community. CEFs (together with community response persons) successfully traced and located 32,108 (70%) clients and provided feedback to the case manager about the tracing outcome. Finally, case managers notified CEFs when 27,269 (59%) clients returned to care (Supplement 5a).

Likewise, case managers registered a line-list of 152,054 index clients with HIV at the health facility, and they assigned CEFs to elicit their sexual contacts in the community. CEFs and community response persons elicited 375,217 sexual contacts after receiving consent. They offered them counseling, conducted HIV testing for 171,550 clients, and provided HIV self-test kits to 156,549 contacts based on their preference. CEFs provided feedback to case managers about the HIV test result via the UDS. A total of 14,676 (8.6%) individuals newly identified as testing positive for HIV were linked to a health facility for treatment (Supplement 5b).

Moreover, 449,279 clients received referral services including adherence counseling, disclosure support, food support, community psychosocial and spiritual support, family planning, and disease screening. CHWs also sent SMS messages to 4,095 clients as appointment reminders based on their needs.

### UDS Utilization for Routine Data Collection

Frontline CHWs relied on paper-based tools to register client interactions and services at the SDP level. They entered client-level data into UDS during or shortly after service delivery using mobile accounts.

Of the 1,766 mobile accounts opened, 1,628 (92.2%; 95% confidence interval [CI]=90.8%, 93.4%) have submitted data. The maximum number of forms submitted by a mobile account was 29,500 forms. Above site, 368 of the 392 web accounts (93.9%; 95% CI=91.0%, 96.0%) have been used by implementation managers or monitoring and evaluation experts to access the dashboard, monitor CHW activity, and review performance.

### Data Quality

In UDS, data quality encompassed timely data access, availability, data completeness, data validity, and other aspects. Various measures were put in place to collect high-quality data. Over 1,210 end-users were trained on UDS, of whom 994 (82%) were frontline CHWs, 140 (11.6%) were monitoring and evaluation staff, and 76 (6.3%) were program implementation managers. In addition, 111 routine data quality assessment visits were conducted, mainly for data verification and site-level technical assistance to CHWs.

### Data Access and Availability

Previously, local implementing partners required at least 1 month to access aggregate data from CHWs, followed by 5 more days for manual report submission via the DATIM data entry user interface. With UDS, however, all community-based implementing partners have daily access to client-level data, and data automation provides readily available aggregate reports for data validation tests and electronic import into the DATIM system within half a day. Table 3 shows UDS data access at various levels.

**TABLE 3.** UDS Data Access at Various Levels, Ethiopia, 2024

Level	Data Access
Level 1	Frontline CHWs at the SDP level collect and enter client-level data into the UDS system. Paper-based records are used as sources of data during RDQA and data verification visits. Mobile apps/accounts are used for client-level data collection and entry to the UDS system electronically.
Level 2	Local implementing partners can access “real-time” case data, form data, and configurable reports. Partners can access the UDS database for data cleaning and quality checking. Data are used by monitoring and evaluation and program staff for monitoring CHW activity. Client-level data can be downloaded in .csv file for further analyses.
Level 3	Partners can access the UDS dashboard to track progress and gain insight. Local implementing partners, government officials, and other stakeholders can access KPIs to facilitate data use for planning (target setting), review performance, and make data-informed decisions. Data validation and testing can be completed prior to DATIM data import (for report submission to the next level).

Abbreviations: DATIM, data for accountability, transparency, and impact; RDQA, routine data quality assessment; UDS, Unified Data System.

**FIGURE 4:**
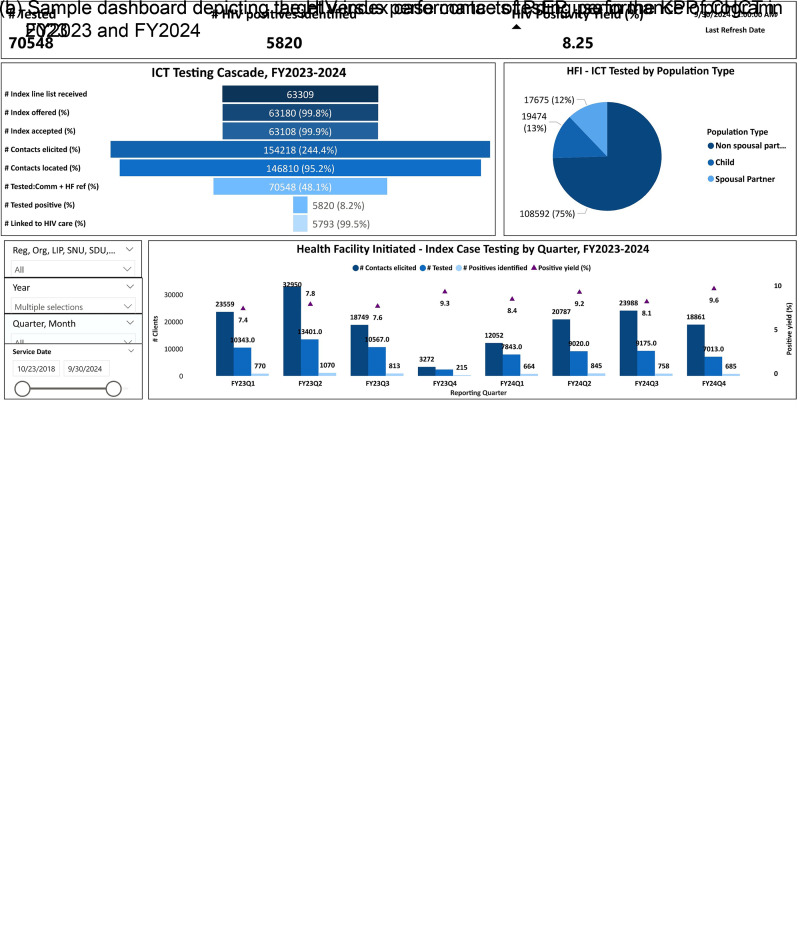

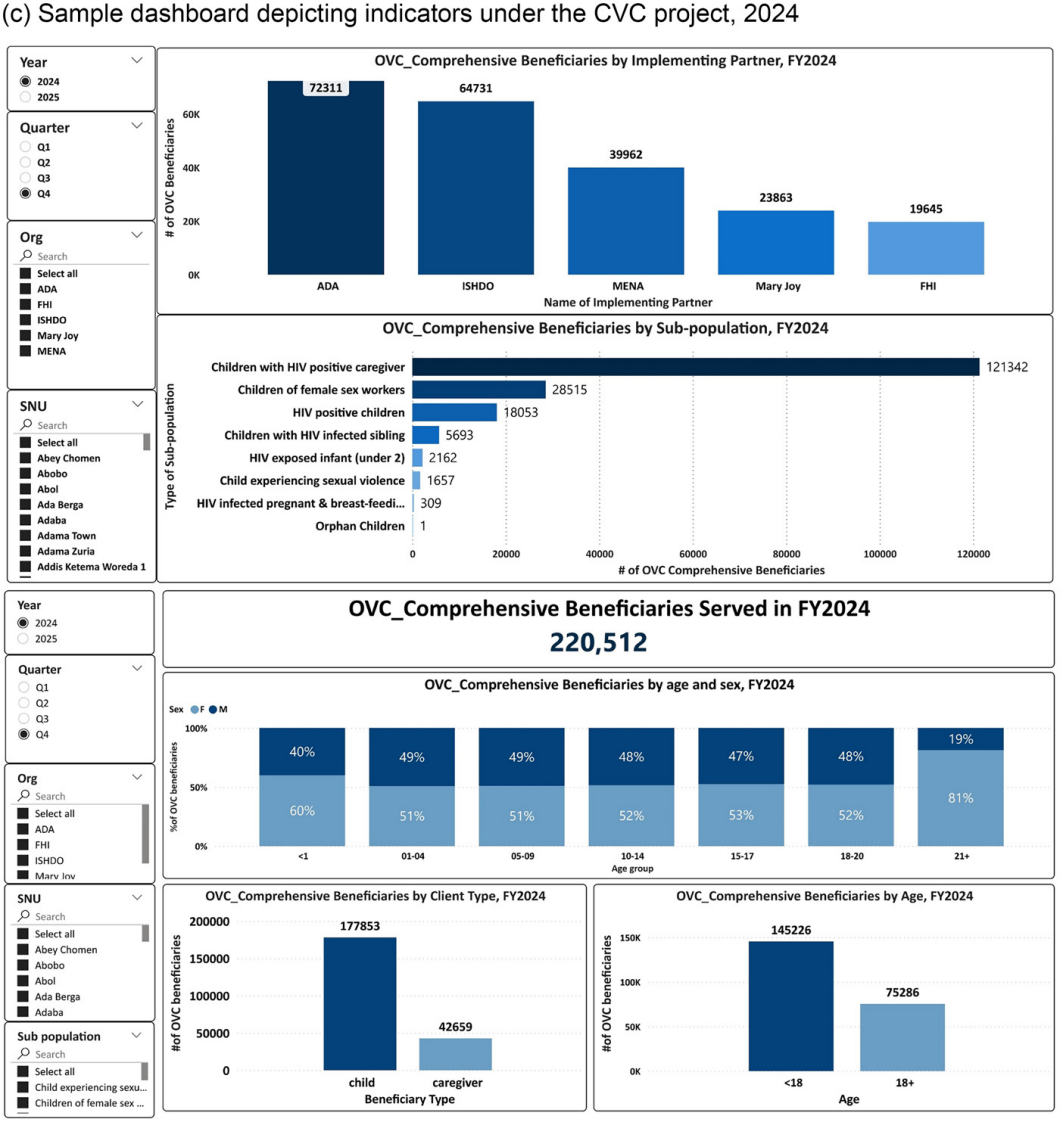
Interactive UDS Dashboard Visuals Depicting Key Performance Indicators in (a) CHCT, (b) KPP, and (c) CVC Projects, Ethiopia, 2024

Developers built an interactive dashboard of KPIs on Power BI to view a quantitative summary of 72 performance indicators, of which 35 (48.6%) belonged to CHCT, 24 (33.3%) to KPP, and 13 (18.1%) to CVC. More than half (n=40; 55.6%) of the indicators showed target versus achievement or the total number of beneficiaries disaggregated by age, sex, service type, or geographic location. Sample dashboard visuals with indicators are shown in Figure 4.

### Data Completeness Composite Score

**FIGURE 5:**
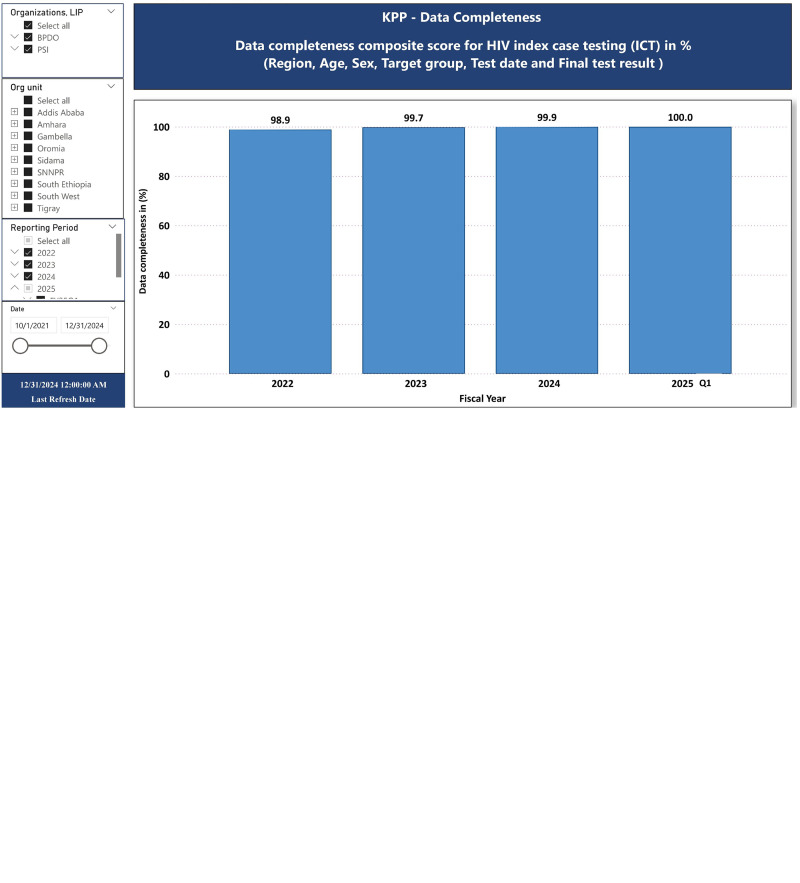
Data Completeness Composite Score Dashboard for Index Contact Testing, Ethiopia Abbreviations: CHCT, community HIV care and treatment activity; KPP, comprehensive HIV prevention, care, and treatment for key and priority populations.

The data completeness composite score showed nearly 100% every year for index case contact testing of the KPP program (between 2022 and 2025) and for key HIV testing indicators and disaggregation variables in the CHCT activity (between 2020 and 2025) (Figure 5).

### Data Validation Checks

Developers carried out data validation checks quarterly using the DATIM validation tool prior to reporting to the next level by implementing partners.^40^ Validation test results for data collected between July and September 2024, for example, showed no invalid data elements or errors.

### Data Use

Six prime local implementing partners, 25 sub-recipient local implementing organizations, 3 technical assistance partners, and numerous stakeholders utilized the UDS dashboard to develop country operational plans, set targets, monitor service delivery, review performance, check data quality, develop reports, and make data-informed decisions. Over 102 performance review meetings were held virtually or in-person to facilitate learnings and to share experiences among local implementing partners, end-users, and stakeholders toward achieving their annual targets. Developers, monitoring and evaluation experts, program managers, and local implementing partners used UDS data to develop 34 abstracts and presented them at various international conferences.^45^^,^^46^

The HIV testing performance of CHCT is a good example of how the UDS was used to support data-informed decisions. Using insights on HIV testing performance from the UDS dashboard, program managers and monitoring and evaluation experts made a strategic decision to focus testing efforts on high-risk populations including sexual partners of individuals newly diagnosed with HIV, interruption-in-treatment cases, people living with HIV struggling with poor treatment adherence, and sexual contacts of PLHIV having an unsuppressed viral load. This strategic shift to a targeted approach resulted in a sharp, nearly twofold increase in the HIV positivity yield, from 4.9% in FY2020 (1,090 individuals tested positive among 22,443 tested) to 8.9% in FY2024/25 (3,849 individuals tested positive among 43,043 tested) (Supplement 5b).

### End-User Support

UDS developers provided post-deployment technical support to end-users, including help with device management, software maintenance, and application updates. Technical difficulties were addressed both onsite and remotely via phone, e-mail, and virtual sessions. Frontline CHWs and local implementing partners received technical assistance with data entry, cleaning, analysis, visualization, reporting, and knowledge management (Table 4). Developers conducted a total of 644 technical support visits to CHWs and local implementing partners to enhance their knowledge and build their capacity in mHealth utilization. Forty-eight UDS partners’ fora were organized to address implementation challenges and share best practices among stakeholders.

**TABLE 4.** Technical Challenges Commonly Faced by Frontline Community Health Workers and Local Implementing Partners During Initial Rollout of the UDS, Ethiopia, 2024

Technical Issues	Measures Taken
**Frontline Community Health Workers**	
Mobile data outage (internet blackout) in areas with security problems	Increased offline data persistence capability of UDS mobile app
Mobile app dysfunction for data entry	Installed the latest mobile app version
Slow loading of cases in case list (mobile app taking too long to navigate and find clients from the case list)	Divided location hierarchies with >6,000 number of beneficiaries; recommended device specification based on the number of beneficiaries
Duplicated and wrong data entry	Introduced validation logics, conducted data cleaning and data verification visits
Delayed data entry	Developed real-time data entry tracking metrics to track field-level operations by CHWs
Incomplete data entry to CommCare/UDS	Developed data completeness composite score monitoring dashboard
Device loss due to damage or theft	Provided device management training and standard operating procedure to CHWs
End-users had the expectation that the move from paper-based to a fully digitized system would coincide with the initial rollout	Developers clearly communicated with end-users about the time it would take to transition fully from a paper to digital system to set expectations
Other technical issues	UDS partners’ forum was conducted to discuss issues and put solutions in place
**Local Implementing Partners**	
Mobile device misspecification	Developers conducted testing of mobile app performance across SDPs with different number of beneficiaries, and they recommended device specifications based on the test results
Data discrepancy and mismatch between dashboard and case data/form data available in the database	Conducted data cleaning and validation checks
Indicator computation challenges	Joint review of indicator definitions and computational procedures
Lack of some functionalities	New features and functionalities were added, such as automated reporting of activity performance
Sub-optimal data use	Conducted monthly KPI review meetings to promote data use for reviewing performance and monitoring, reporting, and data-informed decision making
Manual data entry burden to DATIM by local implementing partners	Developers implemented DATIM data import for performance reporting electronically
UDS complex data export and usage issues	Provided training, developed user manuals and short tutorial videos on data merging and analysis
Misunderstanding of some MER indicator definitions among partners	The unified UDS dashboard guarantees consistency and accuracy of indicators across programs
Bidirectional referral implementation issues	Upgraded UDS software to track actual referral from designated mobile devices
**Software Developers**	
System integration (with the national e-CHIS)	Developers supported the Ministry of Health in developing and pilot testing of the HIV module within e-CHIS
CommCare Power BI integration performance issues	Implemented custom software layer to solve this issue
Technical glitches and bugs	Provided regular software maintenance
Challenge in tracking dashboard usage performance	Developers built a custom web application that displays Power BI dashboard and tracks usage statistics
Mobile workers failed to enter client-level follow-up data despite service provision	Developers added visual reminders to the UDS mobile app when client follow-up is due

Abbreviations: CHW, community health worker; DATIM, data for accountability, transparency, and impact; KPI, key performance indicator; MER, monitoring, evaluation, and reporting; SDP, service delivery point; UDS, Unified Data System.

A common technical challenge encountered by frontline CHWs was that the mobile application was taking too long for the case list to load or to navigate during case management and data entry. Therefore, developers put two key solutions in place: (a) split locations with >6,000 beneficiaries to reduce the data load, and (b) conducted mobile app performance testing across sites with different numbers of beneficiaries allowing a maximum loading time of 10 seconds (Supplement 6). Table 5 presents recommendations to optimize the mobile application’s performance for CHWs by matching device specifications to the beneficiary load at each service delivery point.

**TABLE 5.** Recommended Device Capacity and Specifications Allowing for a Maximum Loading Time of 10 Seconds, Ethiopia, 2024

No. of Beneficiaries per SDP^a^	Recommended Device Capacity (RAM)^b^	Recommended GPU^b^
≤2,000	2GB	Mali-T720 MP1 GPU
2,001–4,000	3GB	Mali-T760 MP8 GPU
4,001–7,000	4GB	ARM Mali-G71 GPU
>7,000	6GB	Qualcomm Adreno 630 GPU

^a^ Mobile app performance testing was conducted using Tecno Camon CX Air and Samsung Galaxy S-series models.

^b^ Based on testing UDS performance on different types of phones, we observed that “UDS case list loading (rendering)” performance showed an improvement with increased GPU rating and by increasing the capacity of the RAM.

Abbreviations: GPU, graphics processing unit; SDP, service delivery point.

### Interoperability and System Integration

UDS and the national electronic community health information system (e-CHIS) were built on a similar digital platform named CommCare, which suggests potential for interoperability. Whereas UDS supported the provision of essential HIV services to vulnerable groups in community settings, e-CHIS supported the provision of primary health care to the general community by health extension workers in rural areas. Initially, developers held 5 meetings with the Ministry of Health for a phased integration of UDS within the broader e-CHIS. However, this did not happen because of mismatch in the scope of HIV work between PEPFAR-funded community HIV activities (which was relatively broad in scope encompassing CHCT, KPP, and CVC) and Ministry-led HIV service delivery by health extension workers (limited to counseling and referral of clients to nearby health facilities). More importantly, the urban health extension worker-led HIV care and treatment services and e-CHIS have not been rolled out in urban settings where UDS is actively implemented.

Likewise, reporting structures were not integrated nor were they fully digitized. While drop-in centers used UDS data to summarize KPIs for submitting reports into the government health data system (DHIS 2) manually, HIV testing data of the CHCT were reported largely through public health facilities. Moreover, implementing partners submitted narrative reports to regional health bureaus quarterly, and government officials participated in semiannual and annual performance review meetings organized by local implementing partners.

Meanwhile, the UDS developers specifically supported the Ministry of Health in developing and pilot-testing new HIV modules within the broader e-CHIS application. UDS developers also provided capacity strengthening training to the e-CHIS app development team at the Ministry of Health, and they shared the UDS dashboard links with 27 HIV program team leaders and monitoring and evaluation experts across all PEPFAR-supported regions of Ethiopia. The aim was to transfer skills and experiences, and to enhance data use for planning, monitoring, and decision making as a means of sustainability.

## DISCUSSION

In this implementation research, we described the design, development, deployment, and utilization of a mobile application to support frontline CHWs, program managers, and local implementing partners for HIV case management and client-level data collection, analysis, visualization, and reporting in the community settings of Ethiopia. The UDS digital health solution was designed by local developers in consultation with stakeholders. Developers followed participatory and user-centered approaches involving end-users and partner organizations throughout the development process. After deployment, they provided end-users with training, software maintenance, routine technical assistance, and supportive supervision both onsite and remotely. Moreover, updated versions were released continually based on user experience and feedback. These user-centered implementation approaches likely facilitated acceptability, adaptation, and utilization of the UDS by CHWs and partner organizations for HIV case management, routine data collection, and performance management. This was in accordance with previous studies conducted in Ethiopia,^28^ Kenya,^47^ and South Africa^48^ where digital health technologies were used to support primary health care workers, collect clinical information from women living with HIV, and improve HIV case detection and linkage to care and treatment, respectively. However, our experience with the user-center design approach showed a longer implementation period than expected. This is partly due to the broad range of UDS functionalities, from software design and development to its utilization for case management, data collection, and data use. In addition, the UDS was specifically implemented within a development program aimed at facilitating HIV prevention, care, and treatment activities. Developers used this period to address the evolving needs of local partners, accommodate user feedback, manage technical challenges by end-users, and update the UDS continuously for ease of use. Previous studies have also reported extended timelines with user-centered methods.^34^^–^^36^

UDS implementation showed a high degree of utilization, i.e., 92% for data collection by frontline CHWs and 94% for monitoring activities and reviewing performance by implementation managers or local implementing partners. This was higher compared to results from a recent study conducted in Ethiopia,^49^ which revealed 50% utilization of the e-CHIS application by rural health extension workers. The deployment of highly skilled and qualified frontline CHWs and the close follow-up, supportive supervision, and routine technical assistance by developers and implementing partners might explain the observed differences. This was not for strict comparison, however, since the settings and users were different. Whereas UDS-using CEFs, social service workers, nurses, and data clerks were deployed in urban or semi-urban towns with improved access to electric power and internet connectivity for UDS use, most health extension workers using the e-CHIS were working in rural areas with limited access to electricity and internet data.^50^ Our results were comparable with previously conducted studies in Northern Ethiopia^28^ and in other developing countries.^51^ The UDS initiative suggests that implementation efforts should consider the needs of frontline CHWs, include end-users in the design and development process, incorporate user feedback routinely, and provide them with training and technical assistance for high levels of usage. However, cautious interpretation is needed in making a causal relationship between user-centered methods and UDS utilization in the present research.

The degree of UDS utilization by frontline CHWs to support essential steps of the case management procedure^38^^,^^39^ was remarkable. CHWs used the application primarily for client registration and enrollment at the point of care. The client ID enabled CHWs to “uniquely” identify beneficiaries, search for them quickly, and prevent duplicate entries in a community setting where the national ID system as a unique proof of identity is unavailable.^52^ In addition, the system-generated unique ID helped to track individual beneficiaries and health service delivery and helped with data merging, cleaning, and analysis. Previous studies indicated that client registration, both in paper-based and electronic registers in parallel, might create an additional workload for health workers.^53^^,^^54^ However, while paper-based registers were used as a source document for data verification and during power outages in the community settings, the UDS platform has completely avoided, if not replaced, the tedious manual work of aggregate reporting by frontline CHWs and local implementing partners, which was quite cumbersome and time-consuming since it required multiple manual disaggregation by sex, age, geographic location, and program type.^42^ Similar findings were reported in previous studies.^27^^,^^28^^,^^51^

The UDS mobile application was used to conduct client need assessments and to generate the care plan automatically, based on client information entered in the system. This was another useful feature of the UDS as a clinical decision-support tool, and in supporting person-centered^55^^,^^56^ and need-based HIV service delivery to the target population. This is in conformity with the digital health guiding principles outlined in the WHO consolidated guidelines on person-centered HIV strategic information,^55^ the 2023 UN High-Level Political Declaration on Universal Health Coverage,^56^ and the 2018 Declaration of Astana on Primary Health Care.^57^ In the KPP project, CHWs used the UDS to support basic HIV prevention, care, and treatment service delivery to vulnerable and high-risk groups, such as female sex workers, who were often inaccessible by the formal health care delivery system.^58^ In this regard, the UDS played a significant role in connecting community- and facility-based HIV services and addressing the needs of marginalized population groups.^59^^,^^60^ As in previously conducted studies,^19^^–^^21^ the UDS was used to send SMS messages to people living with HIV to remind them of the next clinic visits and appointments for drug pick-up or viral load tests. The other interesting feature of the UDS was its functionality to support referrals and linkage to HIV-related services, for example, through the digital sharing of line-lists and patient health information exchange between facility- and community-based health workers. However, these features or functionalities may require further development and research to ensure the continuity of HIV care and to measure their impact on client health outcomes.^59^^,^^60^

Another core function of the UDS platform was its capability to collect routine client-level data offline. The capability of the UDS to collect client-level data, as opposed to aggregate data collection,^54^ enabled flexibility in indicator computation and disaggregation per PEPFAR requirements.^42^ The UDS platform also served as the central hub for data repository, and the web service facilitated a unified point of data access for downstream processes, such as monitoring performance, reporting, and conducting implementation research.^45^^,^^46^ In addition, the UDS dashboard enabled program managers to gain insights, interpret data trends, and make data-informed decisions for targeted and timely action. Above all, the UDS dashboard supported KPI review meetings among the implementing partners to share implementation experiences and to ensure uniform reporting procedures. All these features of the UDS have great potential to enhance service quality and data use in HIV program management, which is often unavailable or suboptimal in most low- and middle-income countries.^8^^,^^54^

A notable outcome of the UDS was its potential to collect high-quality data by end-users, i.e., frontline CHWs and local implementing partners. UDS helped the local partners with daily data access, and it supported their achievement of nearly 100% data completeness. This is consistent with results from a previous study in Ethiopia, which also reported high data completeness scores ranging between 85% and 100% in the public health facilities.^61^ Furthermore, the data validation tests provided additional quality checks, and the results demonstrated no invalid results or errors indicating a high degree of data accuracy. The high level of data quality observed might have resulted from a combination of factors including automated computation of KPIs by the software and regular data cleaning practices and data verification visits by monitoring and evaluation personnel. Moreover, the data validation rules within the UDS likely prevented incorrect data entry, and training and additional technical support from developers likely helped end-users with the standardization of data management procedures across different local implementing partners.

## CONCLUSION

The UDS digital health initiative in Ethiopia showed the feasibility of designing, developing, and deploying a digital tool, with an observed high degree of utilization in community settings of a low-income country in Africa. In addition, the UDS platform supported frontline CHWs and local organizations with standardized HIV case management, linking health facilities and community-based services, and high-quality data collection, analysis, and reporting. Moreover, UDS supported data use by program managers and local implementing partners for monitoring activities, reviewing performance, and making data-informed decisions.

The UDS implementation had several strengths worth mentioning. It was developed by a multidisciplinary team of computer engineers, information technologists, health care workers, program managers, and monitoring and evaluation experts incorporating feedback from such diverse perspectives. We applied a participatory, user-centered design approach, with developers engaging end-users and stakeholders in the design and development process. Developers incorporated user feedback and provided them with training and routine technical assistance both onsite and remotely, which likely contributed to high levels of usage. The platform and data quality assurance mechanisms helped to collect high-quality program data in a real-world community setting. This may imply the feasibility and adaptation of digital health technologies for case management, high-quality data collection, compilation, and reporting by frontline CHWs and local implementing partners. We used the same software type as the national e-CHIS, which enhances interoperability and paves the way for future integration.

The UDS system also had limitations with identity authentication at the front end, which could be improved further with the introduction of the new national ID system.^52^ However, the system generated ID helped to identify and track individual beneficiaries uniquely. The UDS was not linked with the national HMIS or with the e-CHIS directly. An important limitation is that clients had little or no interaction with the application. The case management was partially electronic, which did not avoid paperwork at the point of care. In areas where there was internet blackout due to security problems, frontline CHWs were unable to synchronize data in real time. However, they managed to send data electronically by taking the mobile devices to places where there was internet service. Furthermore, cautious interpretation is needed in claiming a causal relationship between user-centered approach and the observed high degree of UDS utilization by end users. Finally, there were different implementation challenges at various levels; however, reporting detailed challenges and client health outcomes is beyond the scope of this manuscript.

## Notes

**Peer Reviewed**

**First Published Online:** June 16, 2026.
